# Loss of life years after a hip fracture: Effects of age and sex

**DOI:** 10.3109/17453670903316835

**Published:** 2009-10-01

**Authors:** Peter Vestergaard, Lars Rejnmark, Leif Mosekilde

**Affiliations:** Department of Endocrinology and Metabolism, Aarhus University HospitalAarhusDenmark

## Abstract

**Background** Patients with a hip fracture have a high mortality; however, it is not clear how large the loss of life-years is over an extended observation period.

**Subjects and methods** This was a cohort study involving all patients in Denmark who suffered a hip fracture between 1977 and 2001 (n = 169,145). The survival rate for these patients was compared to that for age- and sex-matched subjects without a hip fracture (n = 524,010).

**Results** There was a substantial degree of excess mortality, with a pronounced variation in age and sex. The absolute number of life-years lost compared to age-matched subjects without a hip fracture was larger in younger subjects than in older subjects (men aged 51–60 years lived 7.5 years less on average while men over 80 years of age lived 3 years less). Expressed as a percentage, however, older subjects had the largest relative loss of expected remaining years of life. Men ≤ 50 years of age lost 18% of their expected remaining years of life, as opposed to men > 80 years of age who lost as much as 58% of their expected remaining years of life. In women, the trend was similar but less pronounced (27% loss in women ≤ 50 years of age vs. 38% in women > 80 years of age).

**Interpretation** A large proportion of the estimated remaining life is lost after a hip fracture, even in younger patients. Prevention may save life years, although not all of the years lost after a hip fracture may be due to the hip fracture per se.

## Introduction

Patients with a hip fracture have an added risk of dying (Jacobsen et al. 1992), especially within the first year after the fracture (Vestergaard et al. 2007b).

Although there have been a number of studies on survival after a hip fracture (Miller 1978, Jensen and Tondevold 1979, Dahl 1980, Kreutzfeldt et al. 1984, Holmberg et al. 1986, Magaziner et al. 1989, Parker and Anand 1991, Jacobsen et al. 1992, Cooper et al. 1993, Schroder et al. 1993, Pitto 1994, Poor et al. 1995, Magaziner et al. 1997, Vestergaard et al. 2007b), little is known about the absolute risk of death in fracture cases compared to controls, about the contribution of age and sex to the change in absolute risk of death following a hip fracture, and about how large a proportion of the remaining years is lost. Although there have been no randomized controlled trials on prevention of deaths through primary prevention of hip fractures, observational studies have indicated that a large proportion of the excess mortality after the hip fracture may perhaps be related to the hip fracture per se, as there was additional mortality even after adjustment for pre-fracture morbidity and co-morbidity (Vestergaard et al. 2007b).

Recent randomized controlled trials on intervention with intravenous bisphosphonates after a hip fracture have also shown that an increase in survival may be obtained with bisphosphonates (Lyles et al. 2007). After 2 years, the survival seen in hip fracture patients was approximately 75% in women and 55% in men as compared to an expected survival rate of approximately 85% in both sexes (Jacobsen et al. 1992). If some age and sex strata are especially prone to large losses of expected remaining lifetime, prevention may be especially targeted towards these groups. Most studies have limited their observations to the first few years after the hip fracture, i.e. little is known about the long-term effects of a hip fracture on survival (Parker et al. 1991, Jacobsen et al. 1992, Cooper et al. 1993, Poor et al. 1995, Magaziner et al. 1997, Vestergaard et al. 2007b).

Two research groups have reported long-term survival after a hip fracture. The first group (Elmerson et al. 1988) reported a high 10-year mortality rate, especially in patients who were discharged to an institution. The second group (Holmberg et al. 1986) also reported high 6-year mortality, especially in patients discharged to an institution.

Adjustment for co-morbidity in previous studies has had little effect on the excess mortality (Vestergaard et al. 2007b). Absolute rather than multiply-adjusted estimates of lost life-years would thus provide an easily accessible estimate of loss of life after a hip fracture. We assessed the absolute mortality after a hip fracture and compared it to that in subjects with no hip fracture, stratified by age and sex, and determined the absolute number and percentage of remaining years of life lost in patients who sustained a hip fracture over an extended observation period.

## Subjects and methods

In Denmark, the widespread existence of registers covering contacts with the health sector offers a good opportunity to carry out studies on the occurrence of fractures (Frank 2000). The Danish National Health Service provides tax-supported healthcare for all inhabitants, allowing free access to general practitioners and hospitals. Using the unique 10-digit civil registry number that is assigned to all Danish citizens, a complete picture of hospital contact can be established for each individual, and linkage of data between population-based registries can be obtained.

In Denmark, the National Hospital Discharge Register covers all contacts with hospitals, either on an inpatient or on an outpatient basis (Andersen et al. 1999). The register was founded in 1977, but outpatient records were incorporated completely starting from 1995. The files of this register include information on the civil registry number of the patient, the date of discharge, and the diagnoses at discharge (both principal diagnoses and additional diagnoses)—which were assigned only by the physician at the time of discharge according to the Danish version of the International Classification of Diseases, eighth revision (ICD-8), until the end of 1993, and to the Danish version of the International Classification of Diseases, tenth revision (ICD-10). The register has nationwide coverage of public hospitals with an almost 100% completeness of recording and a high precision of diagnoses (Andersen et al. 1999), especially of fracture diagnoses (Mosbech et al. 1995). This cohort study was performed using the whole of the Danish population, which was approximately 5.3 million individuals during the study period.

The study was subject to control by the National Board of Health and the Danish Data Protection Agency.

### Study design

The study was designed as a classical historic cohort study where exposure was presence of a hip fracture or not, and outcome was mortality. Several designs may be chosen for comparison of survival in patients and referents. In this study, we chose a matched cohort design to ensure the presence of referents in all age groups, even the oldest (in other study designs, few older subjects may be chosen if a random sample of the population is drawn). The design was also chosen to allow direct comparison of subjects with and without a hip fracture to allow individual-based assessment of differences in survival based on a large section of the population.

### Identification of hip fracture patients

Data on all patients with a fracture of the hip (ICD8 codes 820.00 and 820.01, ICD10 codes S72.0 and S72.1) in Denmark in the 25-year period between January 1, 1977 and December 31, 2001 were retrieved from the National Hospital Register (Andersen et al. 1999).

### Identification of subjects with no hip fracture

Using the Civil Registration System, which as of 1968 has had electronic records on all changes in vital status, including change of address and date of death for the entire Danish population, we randomly selected 3 subjects with no hip fracture (controls) for each hip fracture patient, matched by sex and year of birth. The controls were selected using the incidence-density sampling technique (Wacholder et al. 1992), that is, the controls had to be alive and at risk of fracture at the time the hip fracture in the patient corresponding to the controls was diagnosed.

The controls were not required to have been hip or femur fracture cases in the period from 1977 through 2001. Controls who later sustained a hip fracture were censored at the time of their later hip fracture.

### Information on vital status

We retrieved information of migrations and date of death from the Central Person Register (via the National Bureau of Statistics). Patients or controls who emigrated were censored on the date of emigration using individual data on observation time.

### Statistics

Data from the different registers were merged at the National Bureau of Statistics, and for each subject the 10-digit civil registry number was substituted by a unique case number; thus, as investigators we had no access to personally identifiable information. Statistics Denmark holds a key to translate the scrambled identification numbers into the original personal identification numbers, thus allowing identification of the individual patients. Mean (SD) was used for descriptive statistics. Survival was analyzed using actuarial methodology.One-year intervals were used for the calculations. The number of life years was calculated as the area under the curve for up to 25 years of observation. The loss of life years was then taken as the difference between the area under the curve for the hip fracture patients and the controls. Comparisons of survival were based on log-rank test. Analyses were performed using Stata version 8.1 (StataCorp LP, College Station, TX) and SPSS version 15.0 (SPSS Inc., Chicago, IL), both in the Unix version.

## Results

Data from 169,145 hip fracture patients and 524,010 subjects with no hip fracture were used in the study. They had a mean age of 77 (64–90) years, and 28% were men, similar between cases and controls (Table 1). Although there were more co-morbid conditions in hip fracture patients, the difference was limited in absolute terms (e.g. was the difference between prevalence of any liver disease statistically significant, although in percent the difference ranged from 1% to < 1%).

**Table 1.** Baseline characteristics of the fracture patients and the control subjects with no hip fracture

Variable	Hip fracture patients (n = 169,145)	Matched controls with no hip fracture (n = 524,010)	p-value
Sex			
Male	46,073 (28%)	144,695 (28%)	–**^a^**
Female	122,422 (72%)	379,315 (72%)	
Age, mean (SD)	77.0 (13.0)	77.0 (13.0)	–**^a^**
Died during follow-up			
Yes	121,953 (72%)	352,636 (67%)	< 0.01**^b^**
Charlson index			
0 (no co-morbidity)	92,571 (55%)	311,823 (60%)	< 0.01**^b^**
1–2	57,579 (34%)	150,835 (29%)	
3–4	13,838 (8%)	39,104 (8%)	
≥ 5	5,157 (3%)	22,248 (4%)	
Co-morbidity before the hip fracture			
Cancer	20,120 (12%)	75,177 (14%)	< 0.01**^b^**
Previous acute myocardial infarction	9,827 (6%)	43,153 (8%)	< 0.01**^b^**
Arteriosclerosis	6,370 (4%)	16,302 (3%)	< 0.01**^b^**
Chronic obstructive pulmonary disease	12,761 (8%)	30,480 (6%)	< 0.01**^b^**
Cerebrovascular disease	16,641 (10%)	39,077 (8%)	< 0.01**^b^**
Dementia	7,402 (4%)	11,225 (2%)	< 0.01**^b^**
Diabetes	11,179 (7%)	29,154 (6%)	< 0.01**^b^**
Heart failure	13,258 (8%)	44,360 (9%)	< 0.01**^b^**
Any liver disease	2,371 (1%)	3,367 (< 1%)	< 0.01**^b^**
Any kidney disease	11,468 (7%)	24,132 (5%)	< 0.01b
Alcoholism	5,423 (3%)	4,678 (1%)	< 0.01**^b^**
Income in DKR (SD)	86,804 (127,447)	96,162 (135,847)	< 0.01**^c^**
Civil status			< 0.01**^b^**
Widowed	78,178 (46%)	263,455 (50%)	
Divorced	14,426 (9%)	36,815 (7%)	
Married	55,561 (33%)	169,097 (32%)	
Never married	20,973 (12%)	54,580 (10%)	
Other	17 (< 0.1%)	63 (< 0.1%)	
**^a^** No comparisons as data were matched.				**^b^** χ2 test.				**^c^** t-test for 2 samples.			

**Figure 1.**
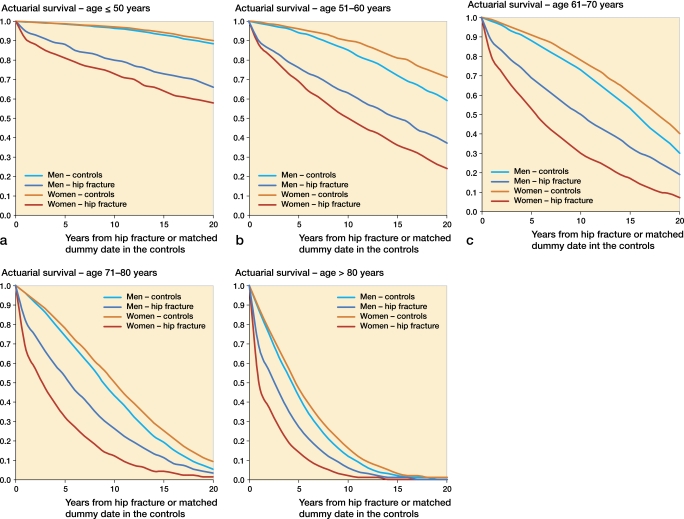
Actuarial survival in patients with a hip fracture and matched controls with no hip fracture, all of whom were ≤ 50 years (a), 51–60 years (b), 61–70 years (c), 71–80 years (d) or > 80 years (e) of age at the time of hip fracture (or matched dummy date in the case of matched controls).

Figure 1 show actuarial survival after a hip fracture, stratified by age and sex. In all scenarios, patients with a hip fracture had a higher mortality than the age- and sex-matched subjects with no hip fracture (2p < 0.01 by log-rank test, and also by Cox proportional hazard regression). The survival curves diverged for more than 1 year following a hip fracture, indicating that there was even excess mortality many years after a hip fracture. In patients aged ≤ 50 years with hip fractures, men had a better survival rate than women (2p < 0.01 by log-rank test) (Figure 1a), whereas in all other age strata women had a better survival rate than men (2p < 0.01 by log-rank test) (Figures 1b–e).

With increasing age, the survival of the subjects without a hip fracture decreased and more subjects died during the first 20 years of observation (Figure 1). However, the survival of the hip fracture patients also decreased. The differences in terms of absolute survival were large, especially within the first year following the hip fracture.

Confounding within age strata did not affect the results. Limiting the analyses to patients who had survived for more than 1 year after the hip fracture (i.e. elimination of the initially high mortality rate) had essentially no effect on the results (data not shown).

Table 2 summarizes the number of life years lost during the observation period of up to 25 years. Whereas the largest loss in absolute numbers of years of life was seen in the youngest age strata, the largest percentage of expected remaining years of life was lost in the older age strata (up to 58% (56–60) of the expected remaining lifetime in men > 80 years of age).

**Table 2.** Observed mean remaining years of life for patients with a hip fracture and matched controls with no hip fracture within the first 25 years after a hip fracture (or corresponding dummy data in the case of controls), years of remaining life lost, and percentage loss of expected remaining years of life; mean (1.96 × SEM)

		Mean remaining years of life			Percentage loss of remaining years of life
Age	Sex	Matched controls without a hip fracture	Hip fracture patients	Years of life lost	
≤ 50 years	M	23 (0.2)	19 (0.4)	4.2 (0.4)	18 (2)
	F	24 (0.2)	17 (0.4)	6.3 (0.4)	27 (2)
51–60 years	M	19 (0.2)	12 (0.4)	7.5 (0.4)	39 (2)
	F	21 (0.2)	14 (0.4)	6.5 (0.4)	31 (2)
61–70 years	M	15 (0.2)	7.5 (0.2)	7.5 (0.4)	50 (2)
	F	17 (0.2)	11 (0.2)	5.6 (0.2)	34 (2)
71–80 years	M	9.5 (0.2)	4.2 (0.2)	5.3 (0.2)	56 (2)
	F	11 (0.2)	6.7 (0.2)	3.8 (0.2)	36 (2)
> 80 years	M	5.1 (0.2)	2.1 (0.2)	3.0 (0.2)	58 (4)
	F	5.6 (0.2)	3.5 (0.2)	2.1 (0.2)	38 (4)

Alcoholism was associated with a loss of expected remaining years. Non-alcoholics had a mean rest life-time of 5.0 (0.4) years, or a mean of 2.6 (0.4) years lost, equivalent to 34% (6)% of the expected remaining life.

## Discussion

In this large-scale population-based cohort study, we have shown a large loss of remaining lifetime in patients with a hip fracture (around one-third of that expected in women > 50 years of age with no hip fracture and more than 50% in men of > 60 years with no hip fracture). A large proportion of the excess mortality took place within the first year following the hip fracture. In contrast to previous studies, our study allows estimation of the percentage of lost remaining lifetime and analysis of long-term survival. The study does not allow calculation of the percentage of the years of life lost that were due to the hip fracture, and thus possibly preventable. Many other factors apart from the hip fracture—such as alcoholism and smoking—may also contribute to the loss of life years, and this should be addressed in detail in future studies.

The expected survival in our study was close to that observed in one previous study: 85% in the study by Jacobsen et al. (1992) as compared to 86% (95% CI: 85–87) in our study. However, the survival rate seen in our study—49% (95 CI: 48–50) in men and 65% (95 CI: 64–66) in women aged ≥ 65 years—was somewhat lower than that observed in the study by Jacobsen et al. (1992) (55% in men vs. 75% in women). The differences may be linked to differences in co-morbidity levels and the fact that we did not exclude pathological fractures linked to cancer metastases.

At 1 year following a hip fracture, Magaziner et al. (1989) reported an expected survival rate of 95% in women and 92% in men as compared to the observed survival rate of 85% in women and 73% in men. In our study, the actual survival rate was 74% (95 CI: 73–75) in women and 60% (95 CI: 59–61) in men, i.e. somewhat lower than reported by Magaziner et al. (1989). The differences may be related to the fact that the population studied by Magaziner et al. (1989) was a selected population from one university hospital in an urban setting.

None of the previous studies have provided follow-up beyond a few years after the hip fracture (Parker and Ananad 1991, Jacobsen et al. 1992, Cooper et al. 1993, Poor et al. 1995, Magaziner et al. 1997, Vestergaard et al. 2007b). Our study provides long-term data showing that it may take more than 15–25 years before the survival in hip fracture patients is in the same range as in the background population—this only being due to the high mortality in the older age classes. Much may thus be gained by prevention.

Previous studies have shown that much of the excess mortality after a hip fracture may be linked to the fracture per se (Vestergaard et al. 2007b), and the primary prevention may thus be of overriding importance (consisting of measures to prevent the hip fracture from occurring at all). If a hip fracture has occurred, prevention of earlier death may prove more difficult and involve several factors such as good operative techniques (Bannister et al. 1990), experience (Lavernia 1998), anesthetics (Davis et al. 1987), and reduction of the time from fracture to surgery (Bottle and Aylin 2006). Furthermore, perioperative and postoperative management with blood transfusion (Halm et al. 2003), and prevention of deep venous thrombosis and pulmonary embolism etc. are also important. As stated above, further studies are needed to clarify how large a proportion of the years of life lost might be prevented.

The primary preventive measures might consist of measures to prevent falls (Becker et al. 2003) and measures to increase bone mechanical competence such as medical treatment with antiresorptive (Cranney et al. 2001, 2002a, b, c, d) , dual-action (O'Donnell et al. 2006), and bone anabolic drugs (Vestergaard et al. 2007a), and of measures to prevent the consequences of a fall e.g. hip protectors (Mosekilde et al. 2006, Parker et al. 2005).

The higher survival rate in men with a hip fracture who are aged less than 50 years than in corresponding women in this age group, which contrasts with the higher survival rate in women in all other age strata, may be related to differences in fracture patterns. In older women the fractures may be related to osteoporosis, whereas in younger individuals accidents may be more prominent, and victims of traffic accidents may be in a better physical condition than elderly subjects suffering from osteoporosis and a number of co-morbidities.

The calculation of life years lost depends on the time span of interest, and may be different for an observation period of, say, one year and 25 years, as in our study. This must therefore be kept in mind when interpreting the data. With a very long observation period as in our study, we covered most of the expected remaining lifetime in the older age categories, and were thus able to estimate the total loss of remaining years of life.

When interpreting the survival curves, it must be remembered that although the curves will eventually converge—as they are limited to the interval from 1 to 0—the mortality conditioned on the survival time may still be different. Although analyses with multiple adjustments were performed, these may not have corrected for all factors.

In this study we addressed the number of life years lost after a hip fracture. Future studies should determine the proportion of life years lost that were in fact attributable to the hip fracture per se. Fractures many years after the hip fracture may not be directly attributable to the hip fracture in itself, but may be associated with the factors that led to the hip fracture. However, if these issues can be addressed, both hip fracture and the life years lost may be prevented.

The strengths of our study are the large sample size, the uniform collection of data, and the long follow-up time. The weaknesses are that individual data on body mass index (BMI) and smoking were not available.
